# Effects of plyometric training on kicking performance in soccer players: A systematic review and meta-analysis

**DOI:** 10.3389/fphys.2023.1072798

**Published:** 2023-04-13

**Authors:** Yeqin Zhang, Danyang Li, Miguel-Ángel Gómez-Ruano, Daniel Memmert, Chunman Li, Ming Fu

**Affiliations:** ^1^ China Football College, Beijing Sport University, Beijing, China; ^2^ School of Psychology, Beijing Sport University, Beijing, China; ^3^ Faculty of Physical Activity and Sport Sciences (INEF), Universidad Politécnica de Madrid, Madrid, Spain; ^4^ Institute of Exercise Training and Sport Informatics, German Sport University Cologne, Cologne, Germany; ^5^ Institute of Physical Education and Training, Capital University of Physical Education and Sports, Beijing, China

**Keywords:** plyometric training, soccer kicking, kicking speed, kicking distance, meta-analysis

## Abstract

This systematic review and meta-analysis aimed to determine the pooled effect size (ES) of plyometric training (PT) on kicking performance (kicking speed and distance) in soccer players depending upon some related factors (i.e., age, gender, skill level, and intervention duration). This study was carried out according to the PRISMA guidelines. Four electronic databases—EBSCO, PubMed, Scopus, and Web of Science—were searched for relevant studies. A total of *n* = 16 studies yielding 17 ES with *n* = 553 participants were finally included in the meta-analysis. A random-effects model was used to calculate Hedge’s *g* with a 95% confidence interval (CI), which showed that plyometric training had a large-sized positive effect on soccer kicking performance (*g* = 0.979, 95% CI [0.606, 1.353], *p* < 0.001). Subgroup analyses were performed according to participants’ characteristics (i.e., age, gender, skill level) and intervention duration, demonstrating no significant differences between these subgroups. The study pointed out that plyometric training is a generally effective method to improve soccer players’ kicking performance, which plays a crucial role in passing and shooting actions during games. As for soccer players and strength and conditioning coaches, the plyometric training aiming to enhance kicking performance has valuable implications in practice. Therefore, besides well-known training methods like power training in the weight room, plyometric training could be incorporated into the overall strength and conditioning programs for soccer players to reach high standards of kicking performance.

## 1 Introduction

Kicking is one of the most important skills in soccer, where different kinds of techniques can be used in offensive and defensive scenarios for the purposes of passing, clearing, and scoring (Peacock and Ball, 2019). Kicking performance is a key skill for the ending actions during games and then has a crucial effect on the game outcome. According to Çobanoglu (2019), 78.98% of the goals in the 2018 World Cup were scored with foot shots. In particular, the goal-scoring odds possibly increase when players try to kick the ball at high speed on this account that the faster kick means less opportunity to be intercepted or saved (Peacock and Ball, 2019). Therefore, it is necessary for the coaching staff to choose an effective way to improve players’ kicking performance. Previous studies examined the influence of different types of training methods on the improvement of kicking performance, including resistance training (Manolopoulos et al., 2013), combined strength and coordination training (Manolopoulos et al., 2006), and eccentric-overload training (Fiorilli et al., 2020).

Over recent years, plyometric training (PT) has emerged as an effective, time-efficient, and easy way to implement training (Asadi, 2013) and improve kicking performance (Ramírez-Campillo et al., 2015a). PT is a classification of strength training exercise consisting mainly of various forms of jumping (De Villarreal et al., 2010; Sole, 2017), which is designed to increase strength and explosiveness, and neuromuscular performance (Wang and Zhang, 2016). Classical plyometric exercises include the drop jump, countermovement jump, squat jump, bounding, and depth jump, which are the natural parts of most sports movements (Asmussen and Bonde-Petersen, 1974; Bauer et al., 1990; Anderst et al., 1994). Depending on the objectives of the training program, different PT exercises are used. These exercises may be used separately or in combination as part of a training regimen.

The identifying feature of PT is the stretch-shortening cycle (SSC) which means the muscle movement involves a high-intensity eccentric contraction immediately after a rapid and powerful concentric contraction (Malisoux et al., 2006). To be specific, an SSC consists o three distinct phases: 1) the eccentric phase, 2) the amortization phase, and 3) the concentric phase (Davies et al., 2015; Sole, 2017). The eccentric phase is characterized by the active lengthening or stretch of the musculotendinous unit. The amortization phase represents the brief time interval between eccentric and concentric muscle action and involves an isometric action. The concentric or propulsive phase consists of concentric muscle action (Davies et al., 2015). The SSC is an indispensable part of PT because it improves the ability of the muscle-tendon unit to produce maximal force within a short time (Komi, 2003), which is conducive to enhancing strength, power output, coordination, and athletic performance (De Villarreal et al., 2010; Asadi et al., 2016; Berton et al., 2018).

To explain the efficacy of PT, a number of mechanisms have been put forth, with varied degrees of emphasis and conclusions drawn throughout the literature. Researchers have noted the importance of elastic strain energy (Kubo et al., 1999; Finni et al., 2001b; Bojsen-Moller et al., 2005; McBride et al., 2008), involuntary nervous processes (Bosco et al., 1981; Bosco et al., 1987), increased active range of movement (Bobbert et al., 1996; Bobbert and Casius, 2005), length-tension characteristics (Ettema et al., 1992; Finni et al., 2001a), pre-activity tension (Kyrölänen et al., 1991), and enhanced coordination along with the innate action of the prestretch (Bobbert et al., 1996; Bobbert and Casius, 2005). In essence, these hypotheses can be classified into three bases: The physiological, mechanical, and neurophysiological basis (Davies et al., 2015). Firstly, the physiologic basis. For the purpose of regulating movement and generating force during PT, the contractile component of the actin and myosin cross bridges with the sarcomere is essential. According to the muscle-tendon unit physiological length-tension curve, the PT makes use of the pre-stretch in order to enhance the ability of the muscle fibers to generate more tension and resultant force production (Davies and Ellenbecker, 1992; Davies and Matheson, 2001). Secondly, a significant factor in plyometric movement is the series elastic components’ (SEC) mechanical behavior (Davies et al., 2015). The SEC stores potential kinetic energy during the pre-stretch motion. As the muscle lengthens to its normal length, the stored energy helps provide concentric force. The term for this is the rebound force reaction. The SEC functions like a spring, with larger forces causing a greater energy release. The elastic recoil of the elastic tissues is thought to be the cause of this impact of PT (Cavagna et al., 1965; Cavagna et al., 1968). The SEC is responsible for between 70 and 75 percent of the muscle’s concentric force increases, making plyometric training very effective (Albert, 1991). Thirdly, the neurophysiological basis. The muscle spindle, the Golgi tendon organ (GTO), and the mechanoreceptors found in ligaments and joint capsules make up the body’s proprioceptors. The facilitation, inhibition, and modulation of both agonistic and antagonistic muscles can result from the stimulation of these receptors (Radcliffe and Farentinos, 1985). Afferent nerve firing increases as the muscle spindle is stretched. The rate of the applied stretch affects how strong of a signal the muscle spindle sends to the spinal cord. The neurological signal produced by the muscle spindle gets stronger the faster it is stretched, which causes a larger contraction of the efferent muscles (the shortening cycle of the plyometric movement). The GTO is another mechanoreceptor that is important for the plyometric stretch-shorten cycle. As a defensive response, the GTO works to keep muscles from contracting too much or becoming excessively tense. As a result, the GTO helps with force modulation during PT. Hence, the goal of plyometric exercise is to desensitize the GTO while increasing the excitability of the neurologic receptors for greater reactivity of the neuromuscular system (Radcliffe and Farentinos, 1985; Ebben et al., 2010). Intense plyometric activities can boost neuromuscular coordination and thus increase neural efficiency. Consequently, by raising the speed at which the muscles can contract, PT improves neuromuscular function. In the end, this technique strengthens the neurologic system to enable greater automatic neuromuscular coordination. (Ebben et al., 2010).

Based on these mechanisms, PT has been implemented to enhance kicking movement, which is an explosive action in soccer (Markovic and Mikulic, 2010; Ramírez-Campillo et al., 2014b). In several studies, the effectiveness of PT has been examined with respect to two dimensions of kicking performance (i.e., kicking speed and kicking distance). Some studies have demonstrated the benefit of PT on kicking speed (Ozbar, 2015; Ramírez-Campillo et al., 2020) and kicking distance (Mengesh et al., 2015; Vera-Assaoka et al., 2020). However, some studies on PT have failed to show any improvement in kicking performance (e.g., Branquinho et al., 2020). Therefore, the potential improvements in kicking performance acquired from the PT intervention remain equivocal compared to regular strength training. As we know, synthesizing results across studies to reach an overall understanding of a problem is an essential part of the scientific process (Gurevitch et al., 2018; Hansen et al., 2022). Consequently, a meta-analytic synthesis is useful for identifying potentially incongruent research findings and promoting evidence-based practice due to its comprehensiveness, quantifiable evidence, and practice-specific recommendations (Gurevitch et al., 2018). Therefore, in order to provide recommendations that are supported by scientific evidence, a thorough examination and meta-analysis of these experimental findings are required in light of the inconsistent outcomes of PT’s efficacy.

In addition, identifying the role of potential moderating factors in the PT effect is also crucial when understanding the overall effect of PT. More importantly, it provides valuable insights for sports practitioners to develop fine-tuned or individual-anchored instruction programs. The effects of PT seem to differ depending on participants’ age, gender, skill level, and duration of PT. For example, regarding age, due to the rapid underlying processes of maturation, it has been discovered that PT has various degrees of effectiveness at different phases of development in youth players (Rumpf et al., 2012; Moran et al., 2017). Furthermore, in terms of gender, as the sex-specific maturational development, researchers have found that the effect of PT varied across gender (Moran et al., 2019). Empirical studies that combine these factors in various ways might sometimes provide contradictory results (Ramírez-Campillo et al., 2016). Therefore, the present study included the participants’ characteristics (i.e., age, gender, skill level) and intervention duration as moderating variables to identify whether the effectiveness of PT would vary according to these variables.

In summary, this systematic review and meta-analysis’s primary purpose was to systematically estimate the overall effect of PT on kicking performance (i.e., kicking speed and kicking distance). The secondary purpose was to determine the variation in PT effect among participants’ characteristics (i.e., age, gender, skill level) and intervention duration.

## 2 Materials and methods

### 2.1 Search strategy

Systematically searches were conducted on four electronic databases (EBSCO, PubMed, Scopus, and Web of Science) until 15 June 2022. All possible keyword combinations from the following two groups were included in the search algorithm: (“plyometric” OR “plyometric training” OR “stretch-shortening cycle”) AND (“kicking speed” OR “kicking velocity” OR “kicking distance”). Additionally, a manual search was performed to find relevant publications in the reference lists of each article that was included. Only peer-reviewed, English-language research articles were included in the search.

### 2.2 Eligibility criteria

The meta-analysis included studies that meet all of the following criteria: 1) population: Healthy soccer players with no previous injury history; 2) intervention: Plyometric training was utilized in the experiment group; 3) comparison: Regular soccer training program was applied in the control group; 4) outcome: Kicking performance was measured, including kicking speed or kicking distance; and 5) study design: The study needed to be a randomized controlled trial.

### 2.3 Literature selection

The studies that met the eligibility requirements were selected independently by two reviewers (YZ and DL). If there were inconsistencies between the two reviewers, they discussed the rationale for their choices, and if one reviewer realized he/she had made an error, then the process was concluded (Devereaux et al., 2002). All disagreements were resolved in this manner.

### 2.4 Data extraction

One reviewer (YZ) gathered the following information from all included studies and entered it into Microsoft Excel. A second reviewer (DL) verified the accuracy of the following data: Author name(s), publication year, title, sample size, participant characteristics (age, gender, and skill level), intervention duration, study design, and kicking performance outcome(s). In the present study, the dominant leg’s kicking performance was chosen as the primary criterion (García-Ramos et al., 2017). If the dominant leg was not specified, the performance with the right leg was chosen (García-Ramos et al., 2017).

### 2.5 Data synthesis

The data formats of the studies included in the meta-analysis are of two types: One data format is pretest-posttest-control design data that includes the means and SDs of the pre-test score and post-test score for the experimental group (plyometric training group) and control group, and the other one is the means and SDs of the pre-test score and the gain score (the post-test score minus the pre-test score) for the experimental group (plyometric training group) and control group.

For the data from the pretest-posttest-control design, Morris (2008) has proposed a reasonable and validated method to estimate effect sizes. The method favored an effect size based on the mean pre-post change in the treatment group minus the mean pre-post change in the control group, divided by the pooled pre-test SD (see Eqs. 1, 2) (Morris, 2008). Then, according to Borenstein et al. (2009), Cohen’s *d* converted to Hedge’s *g* (see Eqs. 3, 4).

For the data from the pre-test score and the gain score with their SDs*,* as the studies included in the meta-analysis provided the pre-test score and its SD, the pooled pre-test SD could be estimated by Eq. 1 (Morris, 2008). In addition, as the included studies provided the mean of the gain score (the post-test score minus the pre-test score) for the experimental group and control group, Cohen’s *d* could be imputed by Eq. 2 (Morris, 2008). Then, the estimations of Correction factor *J* and Hedge’s *g* are the same as the above-mentioned Eqs. 3, 4).

In cases where insufficient data were provided to assess the effect size (ES), attempts were made to contact the study’s authors. For the correlations between the pre- and post-measurements, we followed the recommendations from Follmann et al. (1992) and Higgins et al. (2022) and used an estimated correlation of 0.5. For studies that did not report SDs but provided 90% CIs of means, the 90% CIs would be transformed into SDs, according to Higgins et al. (2022).

$$\left[Pooled\;Pre-test\;SD=\sqrt{\frac{\left(n_{\mathrm{Exp}}-1\right){SD}_{Pre,\mathrm{Exp}}^{2}+\left(n_{Con}-1\right){SD}_{Pre,Con}^{2}}{n_{\mathrm{Exp}}+n_{Con}-2}}\right]$$ (1)

(Morris, 2008)

$$\left[Cohen’s\;d=\frac{\left(M_{Post,\mathrm{Exp}}-M_{Pre,\mathrm{Exp}}\right)-\left(M_{Post,Con}-M_{Pre,Con}\right)}{Pooled\;Pre-test\;SD}\right]$$ (2)

(Morris, 2008)

$$\left[Correction\;factor\;J=1-\frac{3}{4\left(n_{\mathrm{Exp}}+n_{Con}-2\right)-1}\right]$$ (3)

Borenstein et al., 2009

$$\left[Hedge’s\;g=J\times Cohen’s\;d\right]$$ (4)

Borenstein et al., 2009

### 2.6 Data analysis

The primary analysis was performed to estimate the overall effect of the PT intervention on kicking performance by using a meta-analysis based on the random-effects model in view of the heterogeneity within the program of PT intervention, the characteristics of participants, and the measurement of kicking performance. The ES was estimated using Hedge’s *g* with 95% CIs, and its magnitude was determined on the following scale: 0.2–0.5 for small, 0.5–0.8 for moderate, and >0.8 for large (Cohen, 1988).

The *I* ^2^ index was used to determine the degree of study heterogeneity, and the thresholds of heterogeneity for low, moderate, and high were 25%, 50%, and 75% (Higgins et al., 2003). In order to facilitate the comprehension of between-study heterogeneity beyond the existing list of included studies, *τ* ^2^ statistics were also provided (Rücker et al., 2008). To determine the impact of moderating factors (i.e., age, gender, skill level, and intervention duration) on the overall estimate, subgroup analyses using Cochran’s *Q* test and *I* ^2^ were conducted.

Publication bias was evaluated using a funnel plot (Sedgwick and Marston, 2015) and Egger’s regression tests (Egger et al., 1997). Additionally, a sensitivity analysis was conducted by sequentially deleting each study to determine whether the pooled estimate was influenced excessively by a single study (Tobias, 1999).

Meta-analysis and subgroup analyses were carried out with the Comprehensive Meta-Analysis (CMA) 3.0 software (Borenstein, 2022). Two-sided tests were used in all analyses, with a *p*-value of less than 0.05 considered statistically significant.

### 2.7 Study quality assessment

The methodological quality of the included studies was measured using the Physiotherapy Evidence Database (PEDro) scale (Blobaum, 2006). The PEDro scale has 11 items that are used to rate the methodological quality. Each item that is achieved adds one point to the total PEDro score, which ranges from 0 to 10, and item 1 was not included in the study’s methodological quality evaluation (Maher et al., 2003). The quality assessment of the included studies was interpreted as follows: 1–3 points of the total PEDro score equaled poor quality, 4–5 points equaled moderate quality, and 6–10 points equaled high quality (Ramírez-Campillo et al., 2021).

## 3 Results

### 3.1 Study selection

**FIGURE 1:**
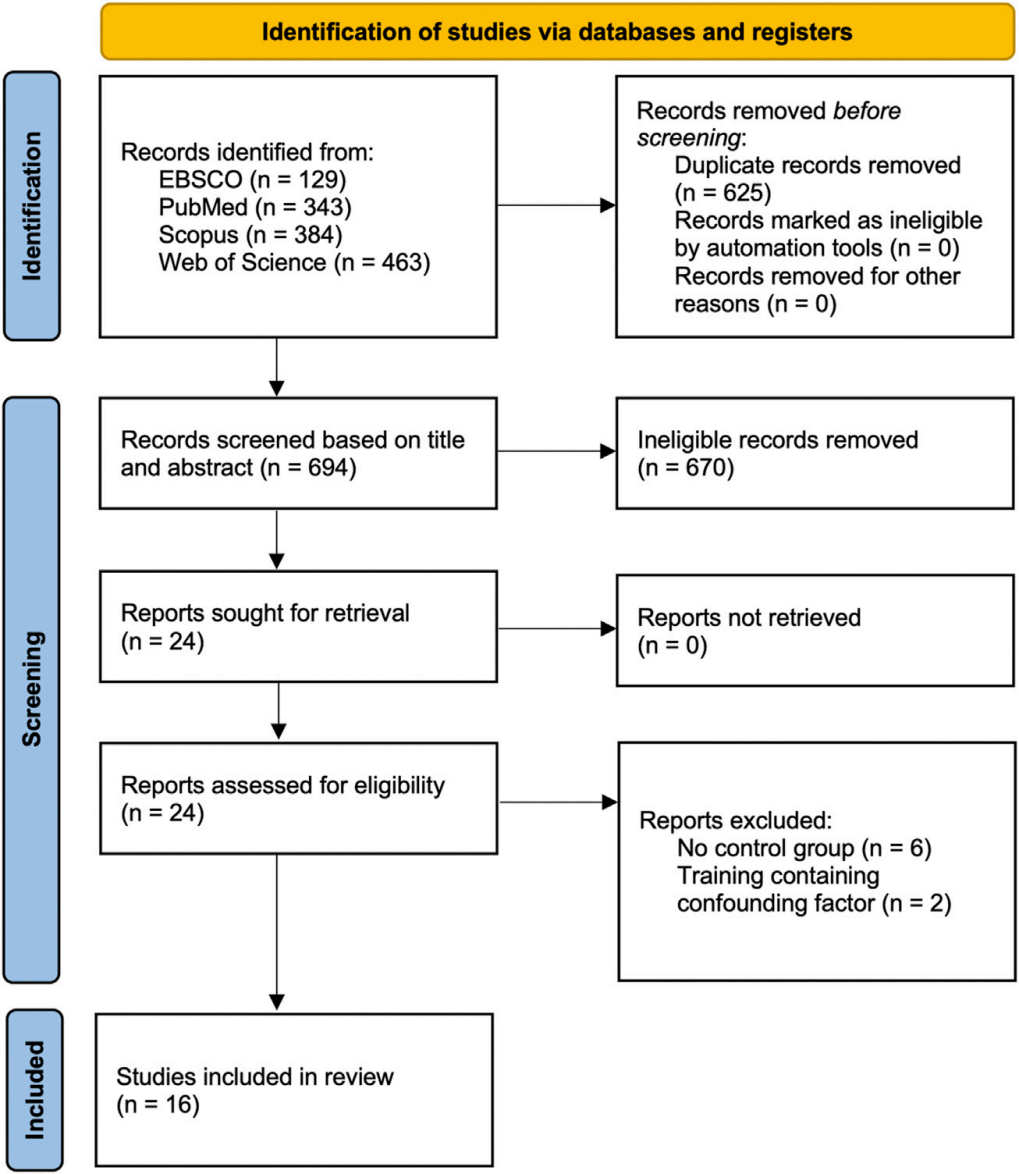
Flow chart of study selection.

The flow chart for the literature selection is shown in Figure 1. The initial search yielded a total of 1,319 records, including 129 from EBSCO, 343 from PubMed, 384 from Scopus, and 463 from Web of Science. After deleting duplicates with 625 records being eliminated, the left 694 records were screened for title and abstract. After screening the title and abstract, 670 records were deleted, and the full text of the remaining 24 articles was examined for compliance with the study selection criteria. Of these, eight articles were excluded. In total, 16 studies were finally included in the meta-analysis.

### 3.2 Study characteristics

The essential characteristics of the 16 studies (17 ES) considered in the meta-analysis are summarized in Table 1. These are all randomized controlled trials. The sample size spans from 15 to 76, totaling *n* = 553 participants. The average age of the participants ranges from 10 to 23 years old. Furthermore, 11 studies focused solely on male athletes, while five studies focused solely on female athletes. In addition, 12 studies recruited non-elite players, while the remaining four used elite players. The intervention duration of the included studies ranges from 6 to 14 weeks. The most common training frequency was two sessions per week (*n* = 12). Regarding kicking performance measures, eight studies have utilized the kicking distance test, and eight have adopted the kicking speed test.

**TABLE 1:** Summary of characteristics of all studies meeting the inclusion criteria.

Study	Sample size (n)	Age (years)	Gender	Skill level	Frequency (per week)	Duration (weeks)	Outcome	PEDro score (0–10)
Mengesh et al. (2015)	EG = 20, CG = 20	20 ± 1.5	F	Non-elite	3	12	KD	5
Ozbar (2015)	EG = 10, CG = 10	19.3 ± 1.6	F	Elite	2	10	KS	4
Ramírez-Campillo et al. (2020)	EG = 8, CG = 7	11–14	M	Non-elite	2	8	KS	9
Ramírez-Campillo et al. (2019)	EG = 19, CG = 20	13	M	Non-elite	2	7	KD	7
Ramírez-Campillo et al. (2018a)	EG = 25, CG = 24	10.9–15.9	M	Elite	2	7	KD	9
Ramírez-Campillo et al. (2018b)	EG = 38, CG = 38	10–16	M	Non-elite	2	7	KD	8
Ramírez-Campillo et al. (2014a)	EG = 13, CG = 14	10.4 ± 2.3	M	Non-elite	2	7	KD	6
Ramírez-Campillo et al. (2015a)	EG = 12, CG = 14	11.4 ± 2.2	M	Non-elite	2	6	KS	6
Ramírez-Campillo et al. (2015b)	EG = 10, CG = 10	10–14	M	Non-elite	2	6	KS	6
Ramírez-Campillo et al. (2018c)	EG = 8, CG = 7	21.4 ± 3.2	F	Non-elite	2	8	KS	9
Ramírez-Campillo et al. (2015c)	EG = 8, CG = 8	13.0 ± 2.3	M	Non-elite	2	6	KS	8
Ramírez-Campillo et al. (2014b)	EG = 38, CG = 38	13.2 ± 1.8	M	Non-elite	2	7	KD	6
Rubley et al. (2011)	EG = 10, CG = 6	13.4 ± 0.5	F	Non-elite	1	14	KD	4
Sedano et al. (2011)	EG = 11, CG = 11	18	M	Elite	3	10	KS	6
Sedano et al. (2009)	EG = 10, CG = 10	23	F	Elite	3	12	KS	5
Vera-Assaoka et al. (2020) Early maturation stage	EG = 16, CG = 16	11	M	Non-elite	2	7	KD	7
Vera-Assaoka et al. (2020) Late maturation stage	EG = 22, CG = 22	14	M	Non-elite	2	7	KD	7

Note. EG, experimental group; CG, control group; F, female; M, male; KD, kicking distance; KS, kicking speed.

### 3.3 Meta-analysis

**FIGURE 2:**
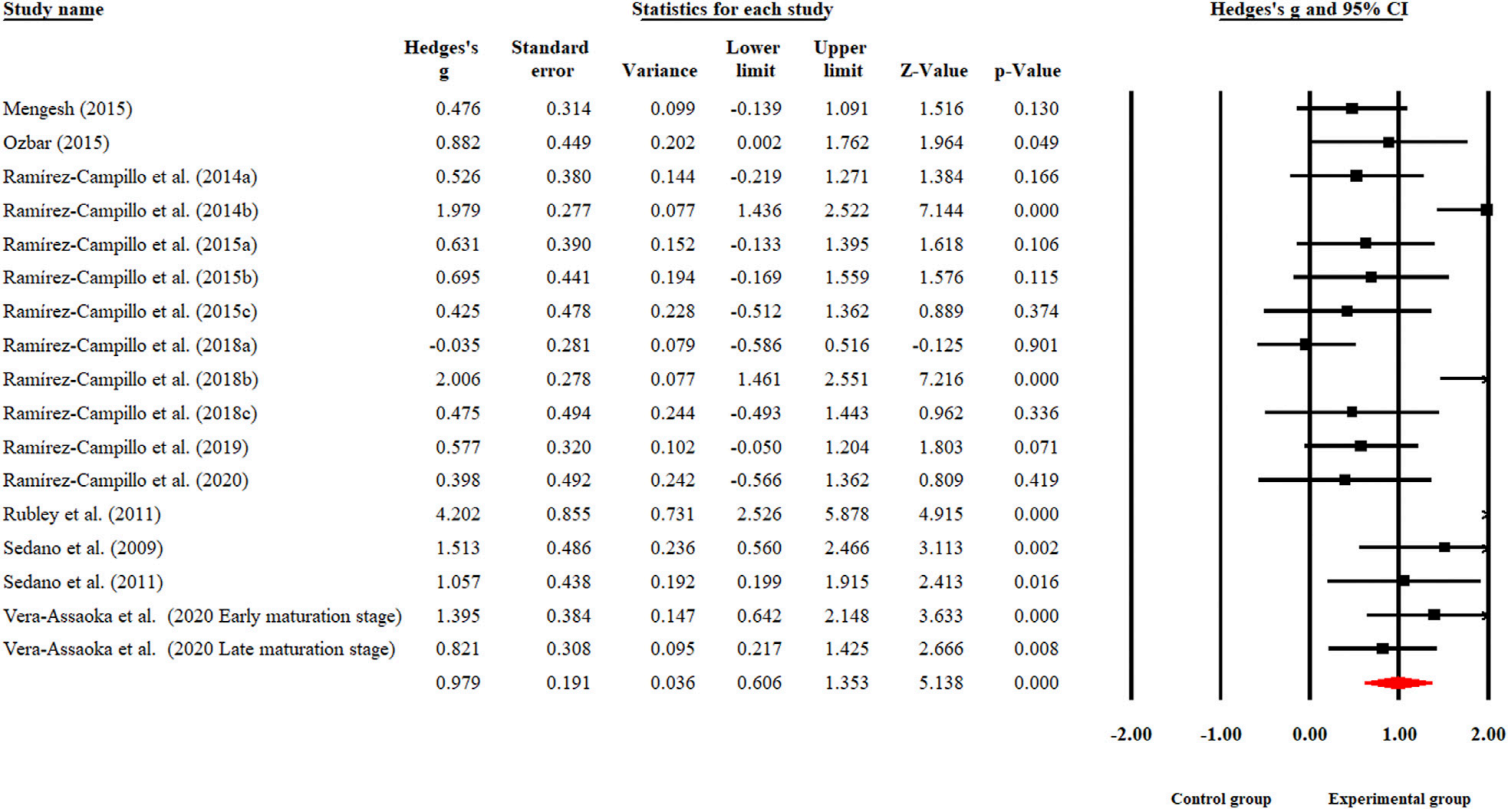
Forest plot of kicking performance in soccer players participating in plyometric training compared to the control group.

Figure 2 depicts the overall estimated ES for the efficacy of PT on kicking performance. There was a large-sized significant enhancement in kicking performance after PT implementation (Hedge’s *g* = 0.979, 95% CI [0.606, 1.353], *p* < 0.001, *τ* ^2^ = 0.449). In addition, a large proportion of the between-study variance was demonstrated according to an *I* ^2^ value of 76.180%.

The findings of the subgroup analysis are presented in Table 2. No significant between-group difference was found when subgroup analyses were conducted based on participants’ age (*Q* = 0.534, *p* = 0.465), gender (*Q* = 0.611, *p* = 0.434), skill level (*Q* = 0.321, *p* = 0.571), and the intervention duration (*Q* = 0.167, *p* = 0.683), suggesting that these variables did not moderate the effect of PT on kicking performance.

**TABLE 2:** Results of subgroup analysis.

Moderator	*k*	Sample size	Hedge’s *g*	95% CI	*p*	*I* ^2^	*Q*	*p*
Age (years)							0.534	0.465
≥18	5	117	0.806	0.438–1.174	<0.001	0.664		
<18	12	436	1.036	0.540–1.532	<0.001	82.307		
Gender							0.611	0.434
Female	5	111	1.299	0.382–2.216	0.005	78.983		
Male	12	442	0.896	0.474–1.319	<0.001	77.138		
Skill level							0.321	0.571
Elite	4	111	0.791	0.062–1.519	0.034	70.335		
None-elite	13	442	1.035	0.604–1.467	<0.001	76.932		
Duration							0.167	0.683
>7 weeks	7	148	1.089	0.446–1.731	0.001	70.353		
≤7 weeks	10	405	0.921	0.439–1.403	<0.001	80.716		

*Note*. *k*, the number of effect sizes; Sample size, the number of participants.

### 3.4 Publication bias and sensitivity analysis

**FIGURE 3:**
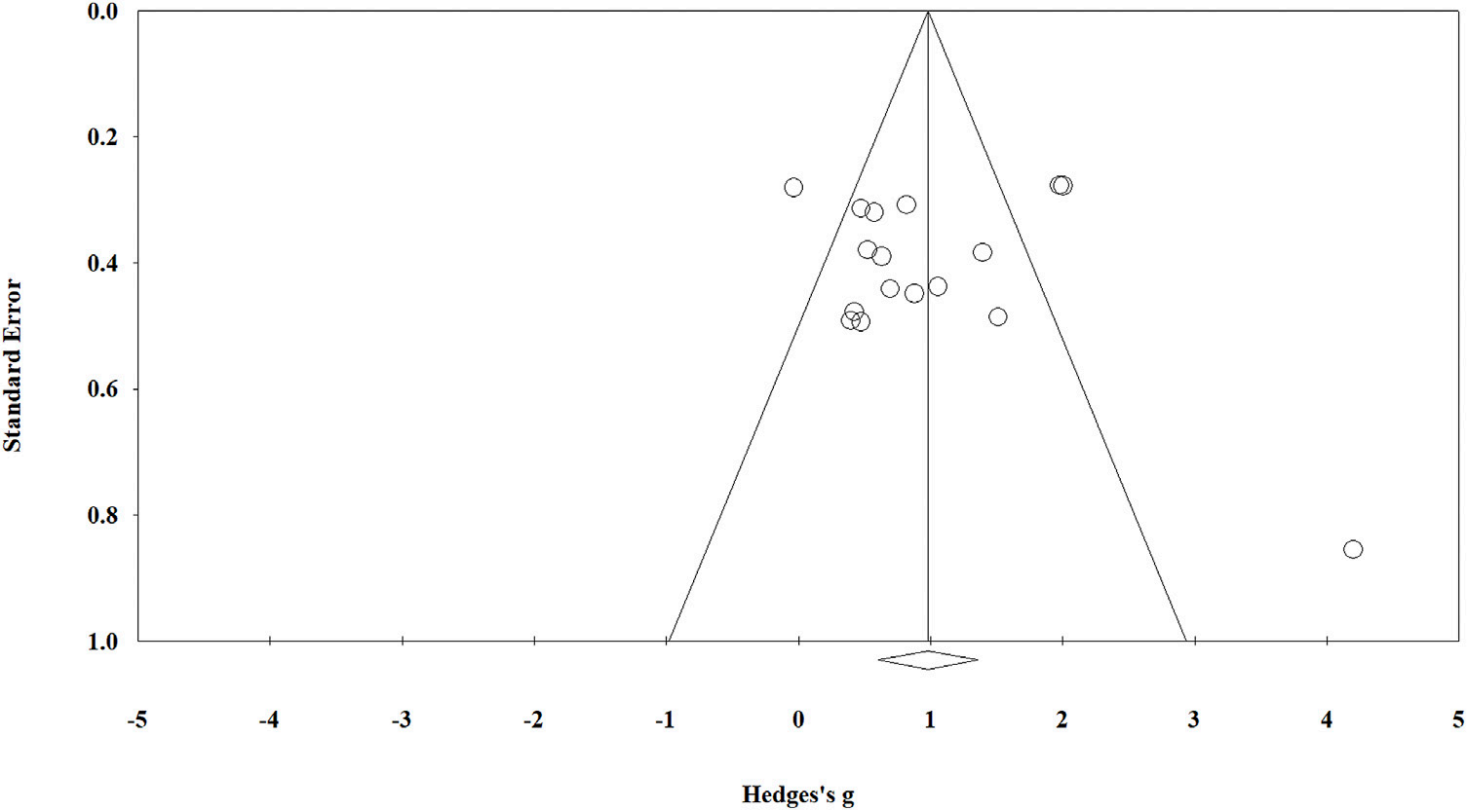
Funnel plot of publication bias.

A visual examination of the funnel plot (see Figure 3) revealed a slightly asymmetrical distribution of the included studies; nevertheless, Egger’s regression test (*t* = 0.332, *p* = 0.745) indicated no significant publication bias. Furthermore, the results of the sensitivity analysis showed no individual study considerably altered the overall effect of PT on kicking performance because when one study was eliminated at a time, the pooled estimate ranged from *g* = 0.880 (95% CI [0.539, 1.221]) to *g* = 1.051 (95% CI [0.688, 1.413]).

### 3.5 Study quality assessment

The total PEDro score of each included study is displayed in Table 1. According to the interpretation of the total PEDro score (Ramírez-Campillo et al., 2021), four studies receiving 4–5 points were deemed to have moderate methodological quality, while the remaining twelve studies obtaining 6–10 points were assessed as being of high methodological quality.

## 4 Discussion

The prime objective of this systematic review and meta-analysis was to determine the effectiveness of PT on kicking performance (i.e., kicking speed and kicking distance). A secondary purpose was to determine the relative significance of various subject characteristics and training variables on kicking performance improvement. The results of this meta-analysis (*g* = 0.979, *p* < 0.001) demonstrated that PT is a practical training protocol to improve kicking performance and revealed the generalizability of PT’s benefits across different subject characteristics and training duration.

### 4.1 The mechanisms of the PT effect on kicking performance

The improvements in kicking performance after PT could attribute to the following hypotheses.

First, PT potentiation is first explained mechanically as the use of elastic energy that has been stored. Our legs, for instance, behave like springs when hopping, jumping, and running; they compress when they make contact with the ground and store energy before rebounding when we push off and release it (Hobara et al., 2008). It is now understood that the tendon serves as the main location for storing elastic energy. (Kubo et al., 1999; Lichtwark and Wilson, 2007). It is theorized that the magnitude of elastic energy stored is proportional to the applied force and the generated deformation (Zatsiorsky et al., 2020). According to previous studies, elasticity significantly improves motor output in sports motions. (Kubo et al., 1999; Lichtwark and Wilson, 2007; McBride et al., 2008).

Second, the stretch reflex and other involuntary nervous system functions have been linked to SSC potentiation (Dietz et al., 1979; Bosco et al., 1981). In a nutshell, the muscle spindles trigger a reflex action upon the musculotendinous unit’s pre-stretching. Type Ia afferent fibers provide a neural impulse to the spinal cord when the muscle spindles notice an abrupt increase in muscle length. The alpha motor neuron and type Ia afferent fibers synapse after that, causing a reflexive muscular movement (Kandel et al., 2000). It is thought that when voluntary and involuntary (reflexive) motions are coordinated at the right time, the agonist’s muscle is activated to its supramaximal concentric level.

Thirdly, according to additional hypotheses, the eccentric action of the prestretch during PT increases the muscle’s active state (Bobbert and Casius, 2005), reducing the electromechanical delay, or the time interval between stimulation and mechanical output, in order to produce force in less time. (Cavanagh and Komi, 1979). As a result, the muscle’s functioning range is expanded, enabling it to produce more force and impulse during the movement’s concentric phase. Moreover, it has been hypothesized that a prestretch and subsequent lengthening would position the muscle in a more advantageous area of the length-tension relationship (Gordon et al., 1966a; Gordon et al., 1966b), increasing force production at the start of and throughout the concentric action as a result (Ettema et al., 1992).

Fourthly, PT could enhance explosive leg power. Kicking is a “whip-like” sequence in which the foot is the quickest link in the open kinetic chain (Carr and Carr, 2004). The velocity of the foot upon impact with the ball is a significant predictor of ball speed and kicking distance (Young and Rath, 2011). As the identifying feature of PT, SSC can assist neuronal and musculotendinous systems in producing maximum force in a minimum time (Markovic and Mikulic, 2010). Bober et al. (1987) have found that maximum knee extension velocity can be 20% higher during a rapid SSC compared to a concentric-only movement in a seated position. Therefore, PT improves kicking performance, possibly by increasing the explosive power of the legs.

Finally, Another possible explanation for PT’s benefits on kicking performance is the enhancement of players’ balance ability after PT intervention. Soccer is a sport that requires bipedal movement, which means standing on one leg while kicking the ball with the other leg (Bressel et al., 2007). That means the player must keep the balance at high speed while kicking the ball powerfully. A growing body of research demonstrated the significance of balance on kicking performance. The research of Cerrah et al. (2016) showed that the kicking velocity of the dominant leg correlated with the dynamic balance ability of the dominant leg and both legs. Furthermore, PT training has shown its advantages in improving players’ balance performance (Ramírez-Campillo et al., 2015a; Alikhani et al., 2019). Therefore, the PT intervention may improve soccer players’ kicking performance by increasing players’ balance ability.

The subgroup analyses show that potential factors (i.e., age, gender, skill level, and intervention duration) do not play a moderating role in the PT effect. Those results may be because of the uneven covariate distribution (e.g., five ES related to participants ≥18 years old vs. 12 ES related to participants <18), implying that the subgroup analyses may not be able to provide accurate results (Richardson et al., 2019). In addition, the unequal number of participants presenting data to these subgroups (e.g., *n* = 117 participants in the subgroup of age ≥18 years old vs. *n* = 436 participants in the subgroup of age <18) suggests that the analyses may not be capable of uncovering subgroup differences (Richardson et al., 2019). It would be prudent for more research to be conducted in the research field in order to investigate the subgroup effect.

### 4.2 Practical applications

PT is a recommended method for enhancing kicking performance. To reach a high level of kicking performance, strength, and conditioning professionals may incorporate PT into soccer players’ overall training program in addition to well-known training approaches such as strength and power training in the weight room. Based on different objectives, coaches can select different forms of PT, such as the drop jump, the countermovement jump, the squat jump, alternate-leg bounding, or hopping. In the practical application process, strength and conditioning professionals can use PT alone or in conjunction with other training methods (Sáez de Villarreal et al., 2015; Bouteraa et al., 2020).

### 4.3 Limitation

Some potential limitations of this study must be outlined. First, because the majority of the studies examined had small sample sizes, the impact of PT may have been overestimated compared to research with larger sample sizes. The effectiveness of PT on kicking performance should thus be examined and replicated using larger sample sizes to boost statistical power. In addition, the binary classification of the continuous data (e.g., the intervention duration > 7 weeks vs. ≤ 7 weeks) may lead to residual confounding and decreased statistical power (Altman and Royston, 2006). Finally, although the included studies did not specify any side effects associated with the PT intervention, it is unclear whether the researchers attempted to document all possible adverse events comprehensively. Therefore, researchers should provide more details on the possible injury, pain, and potential adverse impacts of PT training in future studies to enhance our understanding of the safety of PT.

## 5 Conclusion

The current study demonstrates that the PT intervention could effectively enhance soccer players’ kicking performance. The benefit of the PT effect reveals generalizability across age, gender, skill level, and intervention duration. To further examine the impact of the PT intervention on kicking performance, more research should be conducted in the research field to examine the effectiveness of PT, and larger sample sizes should be recruited to boost statistical power.
